# Intestinal candidiasis and antibiotic usage in children: case study of Nsukka, South Eastern Nigeria

**DOI:** 10.4314/ahs.v17i4.27

**Published:** 2017-12

**Authors:** Ifeoma M Ezeonu, Ntun W Ntun, Kenneth O Ugwu

**Affiliations:** Department of Microbiology, University of Nigeria, Nsukka

**Keywords:** Intestinal candidiasis, children, antibiotic use, diarrhea

## Abstract

**Background:**

Overgrowth of candida results from factors that disrupt the intestinal microbial balance, such as the use of antibiotics. Unregulated antibiotic use and rampant practice of self-medication in Nigeria, is a cause for concern.

**Methods:**

A total of 314 stool specimens were collected from children <1 to 12 years of age in Nsukka, South Eastern Nigeria and screened for candida species using standard methods. Questionnaires were used to collect relevant information on the participants.

**Results:**

Out of the 314 participants, 31.2% had candidiasis, indicated by growth of ≥10^5^ CFU/ml. Four different species of candida were identified. *Candida albicans* had the highest prevalence (59.0%), while *Candida krusei* had the least prevalence (6.0%). Of the 314 participants, 46.5% had diarrhoea, out of which 58.9% had intestinal candidiasis while only 14.3% of the non-diarrhoeic children had candidiasis. Of 208 participants who had taken antibiotics within three weeks of the study, 42.3% had candidiasis compared to 20.8% of those with no recent history of antibiotic use.

**Conclusion:**

The results of this study showed a high prevalence of intestinal candidiasis among children in Nsukka. Strong associations were observed between the presence of intestinal candidiasis and diarrhoea, age and use of antibiotics (p<0.001).

## Introduction

There is a growing appreciation of the abundance and diversity of the trillions of micro-organisms that live on and within the human body and how they influence human health and disease^1^,^2^. The most heavily colonized organ of the human body is the gastrointestinal tract (GIT); the colon alone is estimated to contain over 70% of all the microbes in the human body^3^,^4^. Bacteria are the predominant colonizers of the GIT but other groups of organisms are present in low numbers^5^. The majority of primary gastrointestinal tract colonizers are friendly bacteria; typical among them is the *Lactobacillus* family. The balance between friendly and unfriendly bacteria in the intestinal tract is so important that having the correct balance is synonymous with good physical health^6^.

Candida species (*C. albicans, C. glabrata, C. tropicalis, C. krusei* etc.), otherwise known as intestinal yeasts, constitute one group of organisms that also inhabit the GIT^7^,^8^,^9^. *Lactobacillus* species and other lactic acid bacteria normally keep the Candida in check by their lactic acid production^10^. The perturbation of the gastrointestinal microbiota by the administration of antibiotics, cortisone medication, birth control pills, chemotherapeutic drugs or consumption of excessive alcohol, destroys the *Lactobacillus* enabling Candida to proliferate in the intestine, a condition known as intestinal candidiasis^8^,^11^. The growth of Candida in the GIT causes the release toxic chemicals into the bloodstream, with attendant multiple effects, especially in children^12^.

Use of antibiotics is by far the commonest cause of erosion of normal beneficial flora leading to yeast overgrowth^13^. There is increasing prevalence of intestinal candidiasis in many parts of the world today, all associated with clinical overuse of antibiotics and in recent times, this has led to increased regulation and restriction of the use of antibiotics, in many parts of the world, especially for children. This therefore raises concern for a country like Nigeria, with little or no regulation of the sale/use of antibiotics, most of which are broad spectrum in action. Furthermore, the increasing association of intestinal candidiasis with late-onset autism in children is a serious cause for concern^14^,^15^. This study was therefore conducted to evaluate the prevalence of intestinal candidiasis in children as relates to antibiotics usage. The specific objectives of the study were: to determine the prevalence of intestinal candidiasis in children between 0 – 12 years in Nsukka, South Eastern Nigeria; to identify the Candida species most frequently associated with the condition; and to determine the correlation between intestinal candidiasis and antibiotics use in the children.

## Materials and methods

### Study area

This study was carried out in Nsukka town of Enugu State, South Eastern Nigeria and conducted between November 2014 and February 2015.

### Study groups and population

A total of 314 children were recruited into the study. Of these, 175 were males and 139 were females. A total of 191 fell in the age bracket 0 – 2 years, 83 in the age group of 3 – 6 years, while the participants in the age bracket of 7 – 12 years were 40. The study was designed as a cross-sectional study (population — based survey) aimed at estimating prevalence (in a one time measurement) and correlating the findings with antibiotic intake among participants. The selection criterion was age. Intake of antibiotics was used to stratify the randomly selected participants into two groups at the point of analysis.

### Ethical considerations

Ethical approval was obtained from the Enugu State Ministry of Health (Ref. No.: MH/MSD/EC/0183) and informed consent was obtained from parents/guardians of the children prior to commencement of the study and sampling.

### Questionnaire

Before the collection of specimens, the parents/guardians of each participant were asked to complete a structured questionnaire that included information on: age, sex, mode of delivery of the child, mode of breast feeding, place of birth, how and why antibiotics were sourced for the child etc. The questionnaire also included questions regarding the educational level and occupations of the parents/guardians, as well as their knowledge of antibiotics.

### Specimen collection

Stool samples were collected from each participant into sterile screw-capped, leak-proof universal containers. The samples were properly labelled by making sure that the serial number on each container corresponded with that on the questionnaire before giving them to the participants. The specimens were transported immediately to the Microbiology laboratory for analysis.

### Isolation and identification of organisms

Candida species were isolated and identified on Brilliant Candida agar (Chromogenic agar CM1003; Oxiod, UK), a selective differential medium for the rapid isolation and identification of Candida species of clinical importance. One gram of each stool sample was emulsified in nine millilitres (9 ml) of sterile physiological saline and agitated to attain homogeneity. Then, a 10 µl aliquot of each homogenized specimen was inoculated onto the surface of an agar plate with the aid of an automatic pipette. This was then uniformly spread using a sterile bent glass rod and incubated at 37°C for 24 – 48 h. Colonial appearance and colour of each isolate was compared with documented reactions on the Brilliant Candida Agar as follows: *Candida albicans*, green; *Candida krusei*, pink; *Candida parapsilosis*, Brown or off-white; and *Candida tropicalis*, deep blue. The different Candida species were further identified using direct wet mount preparation, germ tube test, carbohydrate assimilation and sugar fermentation tests.

### Direct wet mount examination

The stool samples were each examined macroscopically for colour of the specimen, consistency (formed, semi-formed, loose or watery), presence of worms like *Enterobius vermicularis, Ascaris lumbricoides* and segments of *Taenia* (tapeworm) species as well as for the presence of blood, mucus or pus. Then, for microscopic examination, a drop of fresh physiological saline was placed on one end of a clean, dry, grease-free microscopic slide and a drop of eosin stain on the other. Using a piece of stick, a small amount of fresh specimen (especially that containing mucus and/ blood) was mixed with each drop. Each preparation was overlaid with a cover-slip. The preparations were examined using the 10× and 40× objectives with the condenser iris closed sufficiently to give good contrast. The preparation was examined especially for yeast cells, motile *Entamoeba histolytica* trophozoites containing red cells, motile *G. Lamblia* trophozoites, motile *Strongyloides* larvae, and the eggs and cysts of parasites. Concentration techniques (saturated sodium chloride floatation technique and the formol ether concentration technique) were also employed.

### Germ tube test

Using a sterile wire loop, a colony of each suspected Candida culture from a 24 – 48 h culture plate was inoculated into 500 µl (0.5 ml) of human serum in a small test tube and placed in a water bath at 35–37°C for 2 h. Using a Pasteur pipette, a drop of the serum yeast culture was transferred onto a glass slide, and overlaid with a cover-slip. The preparation was examined under a light microscope using the 10× and 40× objectives for the production of germ tubes. Formation of germ tubes was seen as long tube like projections extending from the yeast cells with no constriction or septa at the point of attachment to the yeast cells. The germ tube is indicative of *C. albicans* and *C. dubliniensis*^16^.

### Sugar fermentation test

The fermentation basal medium of Wickerham (1951)^17^ was prepared by adding 4.5 g of powdered yeast extract and 7.5 g of peptone to 1 L of demineralized water. Bromothymol blue was added to give a sufficiently dark green colour. A stock solution of Bromothymol blue was prepared by adding 50 mg of powder to 75 ml distilled water. From the stock solution, 4 ml was added to 100 ml of fermentation basal medium. Two millilitre aliquots of basal medium were put into tubes 12 × 150 mm in size, which contained small (approximately 6 × 50 mm) inverted tubes, sterilized at 121°C for 15 min. During autoclaving, the inserts (Durham tubes) were filled with the liquid medium. One (1) ml of the concentrated, filter-sterilized sugar solution was aseptically added to give a final sugar concentration of 2% (w/v).

Thereafter, 1 ml of sterile water was added to a 24 – 48 h SDA slant pure culture and the cells suspended by stirring with the pipette tip. Using this pipette, each tube of test media was inoculated, including a sugar-free control, with a 0.1 ml of cell suspension. The inoculated tubes were gently shaken to mix the cells, and incubated at 25 – 28°C for up to 28 days. The tubes were shaken and inspected at frequent intervals for accumulation of gas in the insert and colour change from green to yellow, which is indicative of sugar consumption. The results were scored depending on the time taken to fill the insert with gas and the amount accumulating: S (strongly positive), insert filled within 7 days; D (delayed positive or latent), insert rapidly filled, but only after more than 7 days; S (slowly positive), insert slowly filled after more than 7 days; W (weakly positive), the insert is not fully filled with gas (e.g., less than one-third full is often considered weak, whereas greater than one-third full is positive); N (negative), no accumulation of gas in the insert.

### Sugar assimilation test

A yeast nitrogen base (YNB) was prepared and agar was added to form a solid medium. This was brought to boil to attain homogeneity. The medium was then distributed into 20 ml quantities and autoclaved. This was allowed to cool to 40 – 45°C, and 2 ml of the yeast suspension to be tested was added to the molten agar using a sterile pipette. The combination was well mixed and dispensed into sterile Petri-dishes and allowed to solidify. A control was also set up using known yeast to be compared with the unknown yeast. The plate was divided into four using a marker on the outside of its bottom. A few grains of each sugar to be tested were sprinkled on the centre of each part and incubated at 25 – 28°C for 4 – 7 days. The cloudy growth seen around any of the sugars was indicative of assimilation by that yeast.

## Statistical analysis

Pearson Chi-Square (χ2) and Fisher's two-tailed exact test (SPSS version 21) were used for analyses of categorical variables. The regression component of Chi Square controlled confounding variables.

## Results

### The prevalence of Candida species among population sampled

Out of 314 participants enrolled in the study, 186 (59.2%) had Candida species in their stool specimens. Of these, 68 (36.6%) had mixed Candida species. Consequently, a total of 264 isolates were recovered from the samples. Four different Candida species were identified namely, *Candida albicans, Candida krusei, Candida parapsilosis* and *Candida tropicalis*. The four species occurred in the following order: *Candida albicans* (42%) > *C. tropicalis* (30%) > *C. crusei* (16%) > *C. parapsilosis* (12%).

### Prevalence of intestinal candidiasis and distribution of Candida species among cases

Candidiasis was indicated by significant growth (≥ 10^5^ CFU/ml) of Candida on a culture plate. Out of the 314 participants, 110 (35.0%) had candidiasis. *Candida albicans* had the highest prevalence of 59.1%, followed by *C. parapsilosis* (19.1%), then *C. tropicalis* (16.4%), while *Candida krusei* had the least prevalence of 5.5%. Among these cases with candidiasis, 53 (48.2%) had mixed aetiology, that is, having significant growth of more than one Candida species while 57 (51.8%) were singly infected. *Candida albicans* was involved in all the cases with mixed aetiology and occurred with other species in different proportions (Table 1).

**Table 1:** Involvement of different *Candida* species in mixed infections

Primary infecting species	Occurrence in mixed infections (%)	Species isolated with/Number (%)			
		*C. krusei*	*C. parapsilosis*	*C. tropicalis*	*C. albicans*
***Candida albicans***	21 (39.6)	7 (33.3)	5 (23.8)	9 (42.9)	−
***Candida krusei***	3 (5.7)	−	−	1 (33.3)	2 (66.7)
***Candida parapsilosis***	15 (28.3)	−	−	7 (46.7)	8 (53.3)
***Candida tropicalis***	14 (26.4)	2 (14.3)	−	−	12 (85.7)

### Prevalence of candidiasis in participants of different age groups and different sexes

The highest prevalence of candidiasis was found in children less than two years old, with a value of 45.0%, compared with children between 3 and 6 years of age (16.9%) and children between 7 and 12 years of age (25.0%). These differences were found to be statistically significant (χ2 = 22.18; df = 2; p < 0.001). Out of 175 males and 139 females sampled, 70 (40.0%) and 40 (28.8%), respectively, had candidiasis. The difference was however not significant (Table 2).

**Table 2:** Occurrence of *candidiasis* in participants of different sexes in different age groups

Age group (yrs)	Number sampled		Number with Candidiasis (%)		
	Males	females	Males	females	Total
**< 2**	111	80	60 (54.1)	26 (32.5)	86 (45.0)
**3 – 6**	45	38	4 (8.9)	10 (26.3)	14 (16.9)
**7 – 12**	19	21	6 (31.6)	4 (19.0)	10 (25.0)
**Total**	175	139	70 (40.0)	40 (28.8)	110 (100)

χ^2^ = 4.29; df = 1; p = 0.05

### Prevalence of candidiasis among diarrhoeic and non-diarrhoeic children

Of the 314 children enrolled in this research, 146 (46.5%) had diarrhoea, while 168 (53.5%) had normal formed stools. Among the children with diarrhoea 86 participants representing 58.9% had candidiasis while only 24 (14.3%) of those without diarrhoea had intestinal candidiasis (χ2 = 68.33; df = 1; p<0.001; Risk estimate =4.12).

### Association of antibiotics usage with occurrence of intestinal candidiasis

Out of the 314 children sampled, 208 (66.2%) reported recent use of antibiotics (currently on antibiotics or use within the three weeks preceding the collection of samples). Of these, 88 (42.3%) had candidiasis, while only 22 (20.8%) of those with no recent use of antibiotics had candidiasis. Chi-square analysis showed this difference to be statistically significant (χ2 = 14.33; df = 1; p<0.001; Risk estimate = 2.04). Respondents' answers showed that Cotrimoxazole (Septrin) was the most frequently used antibiotic (19.3%) in the sampled population, followed by amoxicillin (18.7%), and then ampiclox (12.9%), while the least used antibiotic was cefuroxime (1.1%). Top on the list of reasons given for use of antibiotics were: Cold, 18.5%; typhoid, 16.9%; stomach ache, 15.1%, diarrhoea, 13.9%; fever, 10.5%; teething, 8.2% and cough, 5.4%.

## Discussion

Candida species, otherwise known as intestinal yeasts, constitute one group of organisms that inhabit the GIT. Overgrowth of these yeasts (intestinal candidiasis), can result from conditions that cause perturbation of the gastrointestinal microbiota, especially those that reduce the population of lactic acid bacteria. Antibiotic use is by far the commonest cause of Candida overgrowth. In Nigeria, antibiotic sale is unrestricted, and virtually every available antibiotic can be obtained over-the-counter, without prescription. Moreover, there is the rampant practice of self-medication among the lower-income class, which is extended even to children.

In this study, 59.2% of the children sampled were colonized by Candida in their GIT. Although there is no established “normal” value of Candida in the GIT, some studies have suggested that normal healthy humans may have Candida numbers in the range of 10^3^ to 10^4^,^7^,^11^. In this study, Candida numbers > 10^5^ was taken as overgrowth (candidiasis). Thus, 110 (35.0%) of the children included in the study had overgrowth (intestinal candidiasis). *Candida albicans* was the commonest colonizing yeast and also had the greatest involvement (59.1%) in the disease situation. Three other Candida species (*C. parapsilosis, C. tropicalis, C. krusei*) were identified from the samples and they had prevalence values of 19.1%, 16.4% and 5.5%, respectively. Among the cases with candidiasis, 48.2% had mixed aetiology, with significant growth of more than one Candida species. In all of these cases, *C. albicans* was involved, as shown in Table 1. The predominance of *C. albicans* over other species recorded in this study is in line with reports from other authors^18^,^19^. The identification of the four Candida species both as ordinary colonizers (occurring even in cases without overgrowth), and disease agents attests to an opportunistic role for these organisms in intestinal candidiasis.

There was a significant relationship (p<0.001) between intestinal candidiasis and age in this study, with children below the age of two years having the highest prevalence of disease. No significance (p>0.05) was found between the disease and sex. These results agree with those of Nkuo-Akenji et al.^20^, who studied the prevalence of *C. albicans*-associated diarrhoea in Cameroon. A significant relationship (p<0.001) was observed between intestinal candidiasis and diarrhoea in this study. The calculated risk showed that children with Candida overgrowth were four times more likely to have diarrhoea than those without overgrowth. The role of Candida and other yeasts in diarrhoea has been widely studied by many authors. Some have suggested that the role of Candida in diarrhoea cases is strong enough for the organism to be assigned the status of an entero-pathogen^18^,^20^, while others maintain that despite the frequent isolation of Candida in cases of diarrhoea, its status remains to be proven^19^,^21^. The strong association observed in this study, tends to suggest an aetiologic role for Candida and supports its status as an important opportunistic pathogen.

The results in this study showed a strong association between occurrence of intestinal candidiasis and use of antibiotics in the children. Children who had recently taken antibiotics before they were sampled were two times more likely to have Candida overgrowth than those who had not taken antibiotics. The use of antibiotics has long been considered to be the commonest cause of erosion of normal beneficial bacteria, leading to Candida overgrowth^20^. Broad spectrum antibiotics, in particular, have been said to enhance fungal colonization and risk for candidiasis by destroying competing bacterial flora^12^,^22^.

Although Candida is a normal inhabitant of the intestinal tract, its presence can be more problematic than is commonly supposed, especially when overgrowth occurs. During overgrowth, Candida produces pseudohyphae that push their way into the intestinal lining destroying cells and brush borders, and sending its toxic metabolic by-products through the intestinal wall into the blood. In recent times, this is being increasingly linked to devastating diseases such as late-onset autism^14^,^15^. This therefore raises a lot of concern for a country like Nigeria, where there is little or no restriction of antibiotics sales and use, as well as a prevailing practice of self-medication. The results from this study therefore should send a serious alarm to both health practitioners and regulatory agencies. The responses of the parents and guardians in this study are testament to the rampant misuse of antibiotics in Nigeria. From the questionnaire responses, the most used antibiotics were broad-spectrum antibiotics such as cotrimoxazole, and ampiclox. Many of the respondents also admitted obtaining antibiotics without doctors' prescriptions and top on the list of reasons for administering antibiotics to the children were: cold, 18.5%; typhoid, 16.9%; stomach ache, 15.1%, diarrhoea, 13.9%; fever, 10.5%; teething, 8.2% and Cough, 5.4%.

From the results of this study, it is evident that there is a high prevalence of intestinal candidiasis among children in Nsukka, Nigeria, which is strongly associated with the use of antibiotics in these children. There is therefore a serious need for restriction of use of antibiotics in Nigeria, particularly in children.
